# Cross-cultural adaptation and reliability of the inventory of vicarious posttraumatic growth and research of its influencing factors: a cross-sectional study

**DOI:** 10.1186/s12912-024-02435-5

**Published:** 2024-10-17

**Authors:** Yitong Cai, Yifei Li, Jie Zou, Jie Zhang, Weixiang Luo, Jingping Zhang, Chaoran Qu

**Affiliations:** 1 Xiangya School of Nursing, Central South University, No. 172, Tong- zi-po Road, Yue Lu District, Changsha, Hunan China; 2 Hepatobiliary Pancreatic Cancer Center, Chongqing Key Laboratory of Translational Research for Cancer Metastasis and Individualized Treatment, Chongqing University Cancer Hospital, Chongqing, China; 3 Hunan University of Chinese Medicine, Changsha, China; 4 Department of Nursing Department,The Second Clinical Medical College, The First Affiliated Hospital, Shenzhen People’s Hospital, Jinan University, Southern University of Science and Technology), Shenzhen, Guangdong 518020 China; 5 Department of Operating Room, The Second Clinical Medical College, The First Affiliated Hospital, Shenzhen People’s Hospital, Jinan University, Southern University of Science and Technology), No. 1017, Dongmen North Road, Luohu District, Shenzhen, Guangdong 518020 China

**Keywords:** Nurse, Vicarious posttraumatic growth, Secondary traumatic stress

## Abstract

**Objective:**

The purpose of this study was to translate the Vicarious Posttraumatic Growth Inventory (VPTGI) into Chinese and to assess its reliability and validity in Nurses, Additionally, it explored the correlations between vicarious posttraumatic growth (VPTG), Secondary Traumatic Stress (STS) and demographic variables.

**Methods:**

The Brislin translation model was used to translate the VPTGI into Chinese. Validity analysis involved exploratory factor analysis (EFA), confirmatory factor analysis (CFA), and assessments of convergent validity, discriminant validity, and content validity. Reliability analysis included split-half reliability, internal consistency reliability, and test-retest reliability. Item analysis employed the Critical Ratio Decision Value (CR) method, item-total correlation method, and reliability change method. Single-factor analysis was conducted to examine the relationship between demographic variables and VPTG, while correlation analysis explored the association between STS and VPTG.

**Results:**

The Chinese version VPTGI demonstrated robust content validity (I-CVI: 0.83-1, S-CVI: 0.97), supported by EFA (KMO: 0.933) and significant Bartlett’s test (*p* < 0.001). Four factors explained 67.82% variance, CFA confirmed the model fit (χ2/df = 2.255, RMSEA = 0.079, IFI = 0.931, TLI = 0.914, CFI = 0.930, NFI = 0.882). The Chinese version VPTGI demonstrated high internal consistency (Cronbach’s α = 0.951), with dimensions’ Cronbach’s α ranging from 0.806 to 0.912. Overall, nurses demonstrated a moderate to low level of VPTG and a severe level of STS. Furthermore, there was a significant negative correlation between STS and VPTG.

**Conclusion:**

The Chinese version of VPTGI demonstrated satisfactory reliability, validity, and factor structure, making it a reliable tool to assess VPTG in Chinese nurses. These findings underscore the importance of promoting VPTG and addressing STS among healthcare professionals. Further research in this area is warranted to better understand and support the psychological well-being of nurses.

**Supplementary Information:**

The online version contains supplementary material available at 10.1186/s12912-024-02435-5.

## What is known?

- Nurses may encounter various negative impacts, such as secondary trauma stress and vicarious trauma, due to their empathetic engagement in clinical work.
- Nurses may also undergo personal growth while handling patients’ traumas, a phenomenon known as vicarious posttraumatic growth.
- Presently, the measurement tools utilized in research on VPTG have not been specifically constructed or validated for assessing VPTG, thus casting doubt on the validity of the study outcomes.
- Some studies confirm a connection between STS and VPTG, but the results are inconclusive.

## What is new?

- This study translated the vicarious posttraumatic growth Inventory (VPTGI) into Chinese and verified its reliability and validity.
- Nurses can utilize the Chinese version of the VPTGI to assess their level of VPTG, while also providing a reference for other helping professionals. Furthermore, it can aid researchers in developing intervention measures to promote VPTG.
- The research findings indicate a negative correlation between STS and VPTG. Furthermore, Nurses’ VPTG levels may be influenced by their participation in psychological trauma courses, as well as factors such as age, marital status, and years of work experience.
- Overall, nurses demonstrated a moderate to low level of VPTG and a severe level of STS.

## Introduction

In clinical practice, nurses frequently employ empathy to establish connections with patients’ needs and suffering, potentially leading them to experience varying degrees of indirect trauma [1, 2]. These traumas can trigger adverse outcomes such as compassion fatigue (CF), secondary traumatic stress(STS), and vicarious trauma (VT) [3–5]. These negative effects not only impose significant work pressure on nurses but also contribute to a range of physiological and psychological impacts, such as insomnia and depression [2]. Additionally, they may lead to decreased nursing quality, reduced patient satisfaction, and increased rates of medical errors and nurse turnover [3, 6, 7]. 

However, beyond the negative impacts, in recent years, researchers have begun to focus on growth resulting from indirect trauma. Some studies suggest the possibility of positive changes as well [8, 9]. Calhoun defined the personal growth and meaning gained through others’ trauma as vicarious posttraumatic growth (VPTG) [10]. Similar to posttraumatic growth (PTG), VPTG manifests as positive changes in self-awareness, interpersonal relationships, and life perspectives for individuals [11]. 

VPTG and PTG differ in context, developmental mechanisms, focus of growth, influencing factors, psychological processes, and outcomes [11]. PTG occurs in individuals who have directly experienced trauma, leading to personal growth through cognitive restructuring, emotional adaptation, and finding new meaning in life. This growth includes changes in self-perception, relationships, appreciation for life, spirituality, and new life possibilities. In contrast, VPTG occurs in helping professionals who are indirectly exposed to trauma through empathic engagement with those they support, resulting in professional growth such as enhanced empathy, a deeper understanding of human suffering, and a renewed sense of purpose in their work [12]. PTG is influenced by factors like trauma severity, personal resilience, coping styles, and social support, whereas VPTG is shaped by levels of empathetic engagement, professional support, self-care, and workplace environment. While PTG focuses on personal development following direct trauma exposure, VPTG emphasizes growth within the professional role, requiring a balance between empathy and emotional boundaries to manage vicarious exposure effectively [13, 14]. In Abel’s study, the exploratory factor analysis of the Posttraumatic Growth Inventory (PTGI) when applied to individuals who experienced indirect trauma revealed that the original five-factor model did not fit the data. Instead, a more interpretable two-factor structure emerged, comprising “Personal Growth” and “Changes in Worldview.” However, the study indicated that this two-factor model was unstable, suggesting that the PTGI may not be a suitable tool for measuring vicarious posttraumatic growth (VPTG) and highlighting the need for a VPTG-specific measurement instrument [11]. Many studies have confirmed that these two concepts are distinct [15–18]. 

Nurses face a high risk of indirect trauma, emphasizing the importance of potential positive growth in their clinical work [19–21]. We conducted a systematic review of nurses’ VPTG and found that there is a certain degree of VPTG within the nurses [22–25]. Existing studies commonly suffer from the limitation of not distinguishing the impact of personal trauma history, direct trauma, and exposure to indirect trauma on growth experiences when using the PTGI to assess VPTG among nurses. This direct application of the PTGI diminishes the effectiveness of measurement results, thus presenting less accurate influencing factors and predictive variables of VPTG. Lack of appropriate measurement tools hampers research on nursing-related issues. However, we have identified a vicarious posttraumatic growth inventory (VPTGI) developed by an American scholar [26]. VPTGI measures the positive psychological changes that helping professionals, such as nurses and counselors, experience through indirect exposure to trauma in their work. Grounded in Cohen and Collens’ (2013) framework, the VPTGI distinguishes VPTG from PTG by focusing on growth through empathetic engagement and the processing of others’ trauma. It is considered a comprehensive, reliable, and valid measurement tool. Nevertheless, further investigation is needed to determine whether this questionnaire is applicable in the context of Chinese culture or if modifications are necessary to enhance its reliability and validity.

The connection between indirect trauma’s negative impact (STS) and its positive counterpart (VPTG) among healthcare workers remains debated [27]. Joseph argues that some stress post-trauma is essential for growth [28]. Understanding this link is crucial. It could help tailor support programs, aiding workers in managing stress and advancing professionally, thus enhancing patient care. Yet, conclusive evidence is scarce. Some studies have indeed demonstrated a correlation between STS and VPTG, but there is inconsistency in the research findings [8, 24, 29, 30]. Moreover, current conclusions rely heavily on VPTG measurements using the PTGI scale, potentially introducing some unreliability.

Therefore, this study aims to systematically evaluate the occurrence of VPTG among Chinese nurses confronting indirect trauma by translating and adapting the VPTGI into Chinese. Specific objectives include: firstly, translating the VPTGI to ensure its validity and reliability within the Chinese cultural context; secondly, conducting a large-inventory survey of Chinese nurses using the translated inventory to comprehend the prevalence of VPTG in their clinical practice; and finally, exploring the STS-VPTG relationship, marking the first investigation in Chinese nurses using a VPTG-specific scale.

## Method

### Study design

To ensure equivalence in terms of operability, semantics, concepts, and measurability between the Chinese and original English versions, this study was conducted in two phases. Firstly, the VPTGI underwent translation and cross-cultural adaptation to generate its Chinese version. Subsequently, the reliability and validity of the Chinese version of VPTGI were examined using a cross-sectional survey method.

### Setting and participants

To ensure sample diversity and the feasibility of the study, convenience sampling was employed in this research. Seven hospitals were selected in three cities across central-western and southern China (Henan, Gansu, Sichuan) from July to September 2023. After the Chinese version of the VPTGI is completed, data collection on relevant influencing factors will be conducted again in October 2023. The survey targeted nurses who met the following inclusion criteria: (1) Currently employed nurses; (2) Holding a valid nursing license; (3) Voluntarily participating and providing informed consent. Nurses meeting the following exclusion criteria were excluded: (1) Those not on duty during the survey period, including those on sick leave, leave of absence, or participating in further education programs; (2) Nurses who had been away from their position for more than six months or were on maternity leave during the survey period. An online survey method was utilized in this study. Researchers designed the electronic version of the questionnaire using the online platform WenJuanXing (https://www.wjx.cn/). Participants voluntarily completed and submitted the questionnaire after providing informed consent.

### Sample size

Classical measurement theory provides guidelines for determining sample size to ensure inventory reliability and validity [31, 32]. For Exploratory Factor Analysis (EFA), a minimum sample size of 5 to 10 times the number of inventory items is recommended, accounting for a 20% invalid response rate, requiring at least 200 samples [33]. Confirmatory Factor Analysis (CFA) also requires a minimum sample size of 200, following structural equation modeling requirements [33]. Additionally, for test-retest reliability assessment, a sample size of 30 is needed, with retesting expected 2 to 4 weeks after the initial survey. Therefore, a minimum sample size of 400 is required for this study. Regarding the sample size for the second data collection, based on the principle of multiple linear regression, the sample size should be at least 10 times that of the VPTG entries [34]. 

### Translation

After obtaining authorization from the original author, Professor Jennifer D. Deaton, we commenced the process of translating the original inventory into Chinese. To ensure standardization, we employed the Brislin translation model to facilitate a forward-backward translation process [35]. The process involved: (1) Forward Translation: Two experienced overseas nursing doctoral students independently translated the original inventory into Chinese, which were then integrated into one version by the research team. (2) Back Translation: One nursing instructor and one medical English instructor independently translated the Chinese version back into the original language. These versions were then integrated into one by the research team. (3) Integration: Researchers and translators compared the forward and back translations, analyzed differences, and revised the Chinese version to ensure accuracy and clarity. The revised version underwent final proofreading and review to ensure quality, resulting in the final Chinese version of the VPTGI.

### Cultural adaptation

To ensure the accuracy of the translated inventory and avoid misunderstandings among respondents, we conducted cross-cultural adaptation. This involved consulting six experts to review and suggest modifications for the inventory items. Experts were invited via email and asked to assess the inventory’s content validity using a Likert 4-point scale. Based on their feedback, we made revisions to address semantic issues and ensure better alignment with Chinese cultural norms.

### Instruments

#### Questionnaire for general information

The researchers designed a survey questionnaire based on the purpose and content of this study, referencing relevant literature. This questionnaire includes basic information such as gender, age, job position, education level, professional title, years of work experience, and department affiliation.

#### Vicarious posttraumatic growth inventory

The VPTGI encompasses 32 items across five domains: Negative Responses (initial emotional and psychological distress), Changes in Worldview (shifts in beliefs and empathy), Creating Meaning to Change Self (redefining self-concept and purpose), Changes in Interpersonal Relationships (enhanced empathy and connections), and Client Progress Impacting Growth (inspiration and growth from witnessing client recovery) [26]. These domains capture the complexities of VPTG, reflecting both the challenges and growth that helping professionals experience through their work with trauma survivors. A 6-point Likert scale was utilized, with 1 indicating “strongly disagree” and 6 indicating “strongly agree”. Items 1 to 5 were reverse-scored, and the sum of all items represents the total score of the inventory. After translation and cultural adaptation, the Chinese version of the Nurse VPTGI will be used in subsequent research to further explore VPTG among Chinese nurses and validate its applicability in the Chinese cultural context.

#### The secondary traumatic stress scale(Chinese version)

The Secondary Traumatic Stress Scale (STSS) was used to evaluate the level of STS. The original version was developed by Bride (2004) and then translated into Chinese by Li et al. (2023), which was authorized by Bride [36, 37]. The STSS consists of 17 items, allocated in three subscales (Intrusions, Avoidance, and Hyperarousal) measuring the intensity of STS experienced in the last 7 days. Items are scored on a 5-point scale, ranging from 1 to 5. Based on the total score, scores less than 28 indicate very little or no STS, scores ranging from 28 to 37 indicate mild STS, scores between 38 and 43 indicate moderate STS, scores from 44 to 48 indicate severe STS, and scores of 49 or higher indicate extreme STS.

#### Statistical analysis

SPSS 25.0 software and AMOS 22 software (IBM Corporation) were used for statistical analysis.

#### Item analysis

This study employed Critical Ratio Decision Value (CR) method, item-total correlation method, and reliability change method for item analysis [38, 39]. The decision value method categorizes inventory based on total scores, with top 27% as high-scoring and bottom 27% as low-scoring. An independent samples t-test compares item differences between these groups, removing items with a CR below 3. The item-total correlation method assesses the relationship between individual items and the total score, usually employing Pearson correlation coefficient. Items with a correlation coefficient below 0.3 may be excluded. The reliability change method assesses if removing an item enhances reliability. An increase suggests inconsistency with other items, prompting the use of Cronbach’s α coefficient.

##### Reliability analysis

This study evaluated the stability and consistency of a inventory through internal consistency, split-half, and test-retest reliability [40–42]. ①Internal consistency reliability evaluates the homogeneity and internal correlation among items in the research instrument. A Cronbach’s α coefficient exceeding 0.7 indicates good internal consistency. ②Test-retest reliability assesses consistency in results when the same respondents complete the same inventory measurement within 2 to 4 weeks. Typically measured using correlation coefficients like Pearson or Spearman, a coefficient of 0.7 or higher indicates good reliability. ③Split-half reliability divides the inventory items into two halves and calculates the correlation coefficient between the scores of the two halves. A split-half reliability exceeding 0.7 indicates good inventory reliability.

#### Validity analysis

Validity reflects how well the measured concepts or attributes align with the actual ones, indicating the measurement’s accuracy and effectiveness. ①Construct validity is verified through exploratory factor analysis (EFA) and confirmatory factor analysis (CFA) [33]. When KMO > 0.70 and Bartlett’s test is significant (*P* < 0.05), CFA is considered appropriate. Principal Component Analysis (PCA) and Maximum Variance Orthogonal Rotation were used to assess the internal structure of the Chinese version of VPTGI. If the common factor accumulation of the inventory can explain more than 40% of the variation, the inventory is considered to have good structural validity [43]. Model fit analysis indicated χ^2^/df < 3, and NFI, IFI, TLI and CFI all > 0.8, RMSEA<0.08 [44]. ②Content validity is assessed using expert consultation, with six experts from diverse backgrounds evaluating the relevance of inventory items to their respective dimensions using a Likert 4-point scale. Scale-level Content Validity Index (S-CVI) and Item-level Content Validity Index (I-CVI) are computed, with typically S-CVI ≥ 0.90 and I-CVI ≥ 0.78 considered acceptable [45]. ③Convergent validity is evaluated using the Average Variance Extracted (AVE), where a higher AVE score indicates better convergence validity (typically AVE > 0.5) [43]. ④Discriminant validity is assessed using the Heterotrait-Monotrait Ratio of Correlations (HTMT), where values below 0.85 or 0.90 indicate good discriminant validity [46]. 

#### Data analysis

We conducted data analysis using IBM SPSS Statistics (version 25.0; IBM, Chicago, IL, USA). Descriptive statistics, including means, standard deviations, frequencies, and percentages, were utilized to analyze demographic information, VPTG, and STS. Pearson’s correlation analyses examined associations between variables, while ANOVA and t-tests assessed sociodemographic differences in VPTG. Pearson correlation analysis was used to determine the relationship between STS and VPTG. All statistical tests were two-tailed (α = 0.05).

#### Ethical considerations

This study was approved by the Institutional Review Board at the Central South University (approval number: E2023110). Prior to the commencement of the survey, the principal investigator contacted the nursing department of the survey hospital, explaining the purpose and nature of the study, and obtained support from the hospital, nursing department, and relevant departments.

## Result

### Characteristics of nurse

 This study collected 603 questionnaires, of which 545 were valid after removing those with incorrect responses. Initially, 270 questionnaires were used for EFA, and 275 for CFA. Among them, female participants accounted for 95.8%, and nurses aged between 31 and 40 years old comprised 48.8% of the sample. A total of 380 nurses reported an average daily working time exceeding 8 h, while 334 nurses had not received training on psychological trauma courses. Table 1 shows the characteristics of the nurse.

**Table 1:** Characteristics of nurses (*n* = 491)

Characteristics		n	%
Gender	Male	23	4.22
	Female	522	95.78
Age (years)	20 ~ 30	195	35.78
	31 ~ 40	266	48.807
	41 ~ 50	65	11.927
	> 50	19	3.486
Department	Internal Medicine	230	42.202
	Surgery	111	20.367
	Obstetrics and Gynecology	26	4.771
	Pediatrics	33	6.055
	Emergency Department	32	5.872
	Intensive Care Unit (ICU)	31	5.688
	Operating Room	19	3.486
	Outpatient Clinic	18	3.303
	Psychiatry	2	0.367
	Oncology	5	0.917
	Other	38	6.972
Marital status	Married	435	79.817
	Unmarried	98	17.982
	divorce	12	2.201
Average daily working hours			
	≤ 8	165	30.275
	> 8	380	69.725
Years of nursing experience	< 1	14	2.569
	1–2	25	4.587
	2–5	73	13.394
	5–10	154	28.257
	10–20	205	37.615
	20–30	52	9.541
	> 30	22	4.037
Received training and education on psychological trauma courses			
	Yes	211	38.716
	No	334	61.284

### Cultural adaptation

The expert panel members conducted detailed analysis and discussion on each item, undergoing two rounds of expert consultations. Based on the reviewer’s suggestions, several cultural adaptations were made to the translation of the VPTGI for Chinese nurses to ensure cultural relevance and clarity. Rephrasing was implemented to improve understanding and cultural sensitivity; for instance, abstract terms like “intrusive thoughts” were expanded to include examples such as “recurring worries about patient safety” to make them more relatable. Emotional expressions were modified to align with the cultural norms of more reserved emotional articulation; for example, phrases that emphasized open emotional expression were softened to reflect a more modest approach. Additionally, some items were localized to reflect specific nursing practices, such as modifying descriptions related to professional challenges to directly address the experience of managing patient care and witnessing trauma. These adjustments ensure that the VPTGI is both valid and reliable within the Chinese cultural and professional context, enhancing its applicability for use with Chinese nurses. See the Supplementary file for the comparison of VPTGI between the original version and the Chinese version.

### Item analysis

 The reliability analysis was conducted three times. The first analysis indicated that after removing items 1 and 4, the Cronbach’s α coefficient of the questionnaire was 0.959, higher than the coefficient before item removal. In the second analysis, after removing items 2 and 6, the Cronbach’s α coefficient further increased to 0.965. In the third analysis, after removing items 3, 7, and 23, the Cronbach’s α coefficient increased to 0.967. Consequently, items 1, 2, 3, 4, 5, 7, and 23 were removed from the original questionnaire. Based on the total scores of the remaining 25 items, the samples were ranked, with the top 27% categorized as the high-score group and the bottom 27% as the low-score group. An independent sample t-test was conducted on the two groups, and the results showed that the CR values of all items were greater than 3, indicating high discriminant validity. The item-total correlation method was used to calculate the correlation between the 25 items and the total questionnaire score, and the results showed that the correlation coefficients of all items were greater than 0.3, demonstrating good item homogeneity. Based on these findings, items 1, 2, 3, 4, 5, 7, and 23 were ultimately removed (see Table 2).

**Table 2:** Item analysis for VPTGI

Item	Cronbach’s α if item deleted	CR	Item-total correlation
6	0.97	-15	0.594
8	0.969	-18.4	0.668
9	0.968	-26.38	0.767
10	0.968	-25.42	0.785
11	0.968	-25.94	0.768
12	0.968	-24.25	0.791
13	0.968	-23.69	0.803
14	0.968	-26.84	0.834
15	0.968	-25.22	0.846
16	0.968	-24.42	0.806
17	0.97	-16.76	0.615
18	0.969	-15.08	0.626
19	0.969	-15.81	0.622
20	0.968	-28.31	0.788
21	0.969	-20.81	0.706
22	0.968	-22.93	0.766
24	0.968	-20.39	0.739
25	0.968	-24.58	0.785
26	0.97	-14.93	0.593
27	0.969	-19.68	0.716
28	0.968	-26.34	0.807
29	0.968	-22.19	0.747
30	0.969	-19.01	0.72
31	0.968	-28.07	0.836
32	0.968	-25.68	0.805
Threshold	< 0.970	≥ 3	≥ 0.3

### Validity analysis

#### Content validity

The results of the content validity assessment by the expert panel showed that the Item I-CVI) ranged from 0.83 to 1, while the overall S-CVI was 0.979.

#### Construct validity

 Upon re-examination, EFA was conducted on the dataset. The analysis revealed a Kaiser-Meyer-Olkin (KMO) test coefficient of 0.943and a Bartlett’s sphericity test χ2 value of 5109.520 (*P* < 0.001). indicating excellent suitability for EFA. PCA and maximum variance orthogonal rotation were employed to examine the structure of the inventory. Four factors were extracted under conditions of an undefined number of factors, which accounted for a cumulative variance contribution of 75.307%. The final version of the VPTGI consisted of 25 items in Chinese. The factor loadings for each item are presented in Table 3.

**Table 3:** Factor loading values for each item of the inventory

Items	Factor1	Factor2	Factor3	Factor4
24	**0.714**	0.375	0.169	0.277
25	**0.551**	0.376	0.384	0.36
26	**0.642**	-0.033	0.131	0.373
27	**0.754**	0.31	0.157	0.171
28	**0.719**	0.304	0.397	0.168
29	**0.702**	0.33	0.297	0.08
30	**0.728**	0.269	0.304	0.091
31	**0.663**	0.333	0.454	0.236
32	**0.714**	0.297	0.373	0.202
8	0.17	**0.602**	0.375	0.278
9	0.2	**0.768**	0.45	0.156
10	0.295	**0.725**	0.396	0.147
11	0.294	**0.72**	0.45	0.077
12	0.33	**0.619**	0.434	0.089
20	0.343	**0.741**	0.142	0.361
21	0.358	**0.615**	0.08	0.46
22	0.433	**0.627**	0.169	0.336
6	0.242	0.398	**0.561**	0.231
13	0.323	0.327	**0.786**	0.255
14	0.367	0.289	**0.762**	0.282
15	0.367	0.359	**0.751**	0.241
16	0.34	0.292	**0.756**	0.298
17	0.249	0.214	0.345	**0.559**
18	0.261	0.197	0.231	**0.799**
19	0.159	0.268	0.24	**0.813**
Cumulative variance contribution rate(%)	23.083	44.16	63.061	75.307

**Fig. 1:**
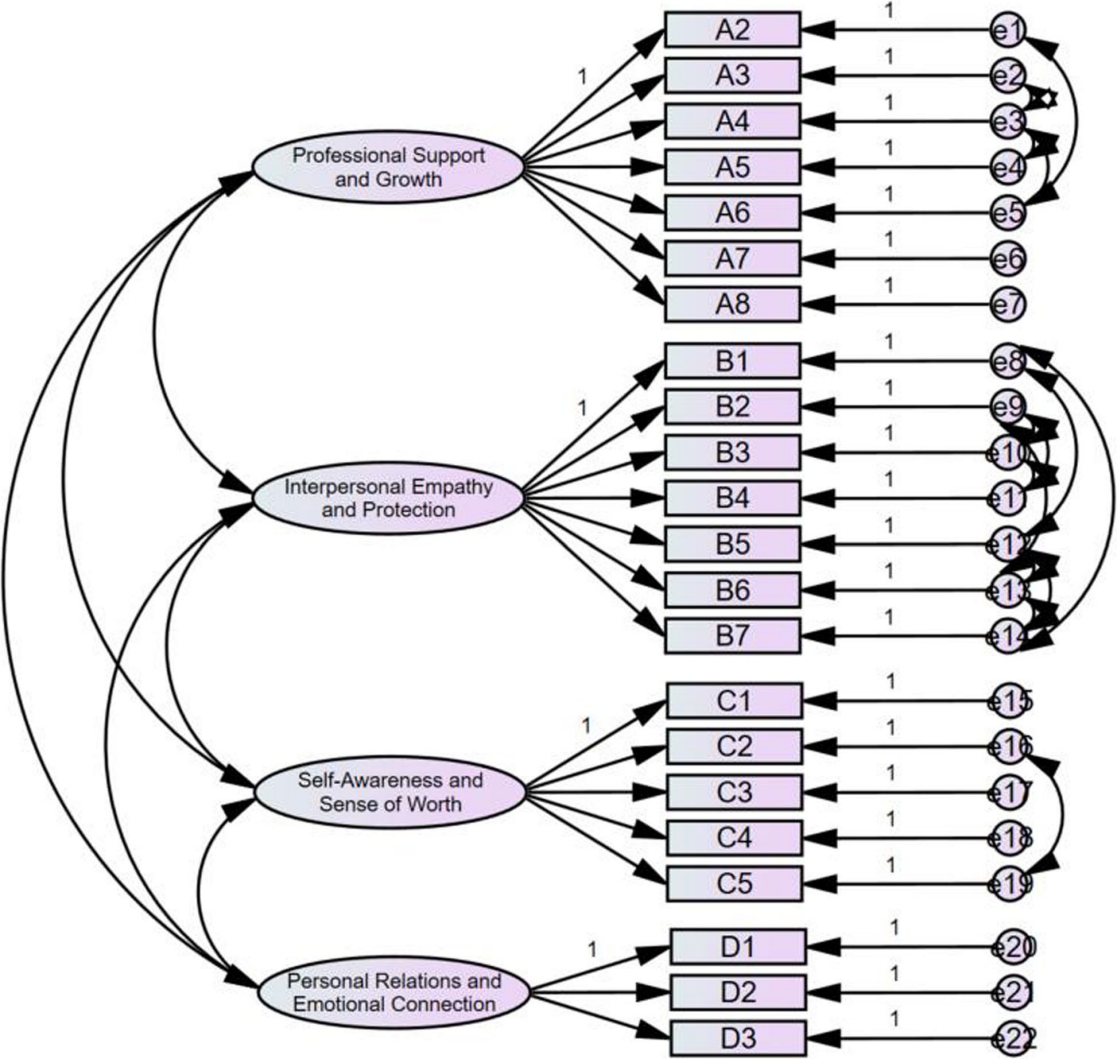
Standardized four-factor structural model of the Chinese version of the VPTGI (*n* = 275)

 A sample of 275 was utilized for CFA to evaluate the fit of the structural model. Using maximum likelihood estimation, 25 items were set as observed variables, and 4 factors were set as latent variables to create the path diagram. During the model modification process, items 32, 24, and 8 were found to have high loadings on multiple factors, indicating that these items may blur the boundaries between different latent variables, thus affecting the discriminant validity of the model. To improve the model’s simplicity and interpretability, these three items were removed. The remaining 22 items were then set as observed variables, and the 4 factors were retained as latent variables to redraw the path diagram. The model fit indices can be found in Table 4, and the results of the CFA are presented in Fig. 1. The initial fit indices did not meet the required standards, so the model was adjusted. After modification, the indices improved significantly, indicating that the Chinese version of the VPTGI has a good model fit.

**Table 4:** Fit results of the modified VPTGI model for Chinese nurses

Indicators	χ^2^/df	RMSEA	NFI	IFI	TLI	CFI
Fit Criteria	< 3.00	< 0.08	> 0.90	> 0.90	> 0.90	> 0.90
Before Modification	4.064	0.106	0.852	0.884	0.868	0.884
After Modification	2.703	0.079	0.909	0.94	0.927	0.94

*χ*^*2*^*/df* Chi-square to degrees of freedom ratio, *RMSEA* Root Mean Square Error of Approximation, *GFI* Goodness-of-Fit Index, *AGFI* Adjusted Goodness-of-Fit Index, *NFI* Normed Fit Index, *TLI* Tucker-Lewis Index, *CFI* Comparative Fit Index

#### Convergent validity

 The AVE estimates for the four dimensions of the Chinese version of VPTGI were 0.607, 0.664, 0.692, and 0.577, respectively. Correspondingly, the Composite CR values were 0.915, 0.933, 0.915, and 0.801. The convergence validity of the Chinese version of VPTGI was deemed acceptable. The Chinese version of the Vicarious Posttraumatic Growth Inventory (VPTGI) for nurses consists of 25 items, divided into four dimensions. Dimension 1 contains 7 items and is labeled “Professional Support and Growth.” Dimension 2 also includes 7 items and is labeled “Interpersonal Empathy and Protection.” Dimension 3 consists of 5 items and is labeled " Self-Awareness and Sense of Worth.” Dimension 4 contains 3 items and is labeled " Personal Relations and Emotional Connection.” Details can be found in Table 5.

**Table 5:** Convergent validity of the Chinese version of the VPTGI for nurses

Factor	Item		Estimate	AVE	CR
Professional Support and Growth	A2	24	0.764	0.61	0.92
	A3	25	0.773		
	A4	26	0.669		
	A5	27	0.752		
	A6	28	0.867		
	A7	29	0.842		
	A8	30	0.772		
Interpersonal Empathy and Protection	B2	9	0.863	0.66	0.93
	B3	10	0.759		
	B4	11	0.801		
	B7	12	0.795		
	B1	20	0.733		
	B5	21	0.815		
	B6	22	0.925		
Self-Awareness and Sense of Worth	C1	6	0.459	0.69	0.92
	C2	13	0.875		
	C3	14	0.926		
	C4	15	0.911		
	C5	16	0.891		
Personal Relations and Emotional Connection	D1	17	0.615	0.58	0.8
	D2	18	0.81		
	D3	19	0.835		

#### Discriminant validity

 Discriminant validity analysis results indicate that there is appropriate discriminant validity among the different dimensions of the inventory. The HTMT values between the dimensions range from 0.764 to 0.849. These findings suggest that the inventory effectively distinguishes between the various dimensions when measuring different concepts or variables, thereby demonstrating good discriminant validity. Further details are provided in Table 6.

**Table 6:** HTMT values for each dimension of the inventory

Dimension	HTMT
A vs B	0.841
A vs C	0.713
A vs D	0.754
B vs C	0.849
B vs D	0.764
C vs D	0.739

A: Professional Support and Growth

B: Interpersonal Empathy and Protection

C: Self-Awareness and Sense of Worth

D: Personal Relations and Emotional Connection

#### Reliability

 The reliability of the Chinese version of the VPTGI. The Cronbach’s α coefficient for the overall questionnaire was 0.965, with Cronbach’s α coefficients for individual dimensions ranging from 0.795 to 0.936. The split-half reliability coefficient for the overall questionnaire was 0.922, with split-half reliability coefficients for individual dimensions ranging from 0.757 to 0.901. A random sample of 30 healthcare professionals was re-evaluated after a two-week interval, and the test-retest reliability coefficient for the overall questionnaire was 0.899, with test-retest reliability coefficients for individual dimensions ranging from 0.717 to 0.899. Detailed data can be found in Table 7.

**Table 7:** The reliability of the inventory and each dimension

Dimensions	Cronbach's α	Split-half reliability	Test–retest reliability
A	0.93	0.901	0.873
B	0.936	0.825	0.776
C	0.92	0.901	0.891
D	0.795	0.757	0.717
Total iventory	0.965	0.922	0.899

A: Professional Support and Growth

B: Interpersonal Empathy and Protection

C: Self-Awareness and Sense of Worth

D: Personal Relations and Emotional Connection

#### Re-analysis

After removing three items based on structural validity, 22 items were re-analyzed. An independent t-test showed CR values above 3 for all items, indicating strong discriminant validity. Scores below 88 indicated low VPTG, and scores above 110 indicated high VPTG. Item-total correlations exceeded 0.3, showing good internal consistency. Reliability analysis showed Cronbach’s alpha for all items was lower than the overall α of 0.965. Content validity showed I-CVI values between 0.83 and 1, with an S-CVI of 0.984. Exploratory factor analysis yielded a KMO of 0.955 and Bartlett’s χ² of 5784.038 (*P* < 0.001), with four factors explaining 76.134% of the variance, confirming strong structural validity.

#### Univariate analyses of the factors associated with VPTG and STS

 For the second round of data collection, a total of 400 questionnaires were distributed, resulting in 379 valid responses. The majority of nurses were female (*n* = 376), with the highest number of nurses aged between 31 and 40 years old, totaling 233 individuals. Most nurses (*n* = 197) reported not having received any psychological training during this period. Single-factor analysis revealed that whether nurses had undergone psychological trauma courses, age, marital status, years of nursing experience could influence VPTG, as detailed in Table 8.

**Table 8:** ANOVA between total VPTG/STS score and demographic variables 379

Variables	Category	VPTG					STS			
		n	Mean	SD	F/t	P	Mean	SD	F/t	P
Gender										
	Male	3	111.67	13.87	0.42	0.52	39.67	7.51	0.32	0.57
	Female	376	106.10	14.83			44.62	15.22		
Age (years)										
	20 ~ 30	83	103.83	15.22	3.97	**0.01**	44.28	15.64	0.27	0.84
	31 ~ 40	233	105.47	14.63			45.03	14.60		
	41 ~ 50	53	112.15	14.02			43.64	16.29		
	> 50	10	109.20	13.16			41.60	19.70		
Department										
	Internal Medicine	151	105.3	14.90	2.02	0.13	45.1	13.78	0.85	0.43
	Surgery	95	104.6	15.57			42.8	13.25		
	Other	133	108.2	14.03			45.3	17.76		
Marital status										
	Married	319	107.1	14.79	4.00	**0.02**	44.6	15.57	0.12	0.88
	Unmarried	52	100.9	14.91			44.3	13.33		
	divorce	8	104.3	7.09			47.1	10.70		
Years of nursing experience(years)										
	< 1	2	108.00	1.41	4.31	**0.00**	35.00	22.63	0.76	0.61
	1–2	10	98.70	9.36			43.10	10.46		
	3–5	18	92.83	16.71			42.83	13.92		
	6–10	108	105.41	14.12			43.50	15.03		
	11–20	193	106.87	14.70			46.04	14.88		
	21–30	37	112.41	14.95			43.00	17.45		
	> 30	11	107.91	10.82			40.82	18.81		
Involvement in the care of terminally ill patients										
	Yes	212	106.61	15.17	0.47	0.49	44.44	15.46	0.04	0.84
	No	167	105.56	14.37			44.75	14.84		
Received training and education on psychological trauma courses										
	Yes	182	108.19	15.29	6.75	**0.01**	43.47	16.08	1.89	0.17
	No	197	104.26	14.14			45.61	14.24		

#### VPTG, STS and their associations

The average score for VPTG was 106.05 (SD = 14.82), indicating a moderate to low level among nurses. For STS, the average score was 44.58 (SD = 15.17), indicating a severe level. Specifically, 15.6% of nurses exhibited low levels of VPTG, while 44.3% exhibited moderate levels. In contrast, for STS, 41.2% of nurses experienced extreme levels, with only 13.2% and 22.2% experiencing no or mild STS, respectively. Detailed information can be found in Table 9. The correlation analysis results indicate a significant negative correlation between STS and VPTG(*r*=-0.021, *P*<0.001).

**Table 9:** Mean and standard deviations of variables

Variables	Mean	SD	Frequency	Percentage
VPTG	106.15	14.82		
Low level			59	15.6
Average levels			168	44.3
High level			152	40.1
STS	44.58	15.17		
None			50	13.2
Mild			84	22.2
Moderate			47	12.4
Severe			42	11.1
Extreme			156	41.2

## Discussion

Nurses, as a high-risk group exposed to indirect trauma, cannot be overlooked for their impact [47]. With the emergence of positive psychology, research suggests that exposure to indirect trauma has both negative and positive effects on individuals [21]. Since the 1990s, positive psychology has garnered increasing attention. However, current research on nurses exposed to indirect trauma tends to focus more on negative outcomes, neglecting the perspective of positive psychology [19]. Some studies indicate that post-traumatic growth can mitigate nurses’ burnout and enhance their job retention [21]. Yet, there is a lack of specific tool to assess post-traumatic growth in China. Therefore, this study aims to translate the empirically validated VPTGI into Chinese, providing a reliable tool for measuring VPTG among nurses in the Chinese cultural context. This effort is crucial for promoting widespread attention and research on VPTG among nurses.

### Cultural adaptation and translation process

To ensure rigor, we strictly followed scale introduction principles and the Chinese translation process [35]. Considering experts’ backgrounds, we invited six with relevant research and clinical experience. This ensures the Chinese VPTGI aligns with our linguistic and cultural norms. Several cultural adaptations were made to enhance the clarity and applicability of the VPTGI for Chinese nurses. For example, abstract psychological terms like “intrusive thoughts” were expanded with culturally relevant examples (e.g., “recurring worries about patient safety”) to make the items more relatable to Chinese healthcare settings. Emotional expressions, which in Western contexts are more direct, were modified to reflect the more reserved emotional articulation typical in Chinese culture. This process ensured that the translated tool was both valid and reliable within the Chinese cultural and professional context.

### Differences in Factorial Construct

The differences between the Chinese and English versions of the VPTGI are notable in the restructuring of the factorial construct. The original English version of the VPTGI comprised three dimensions: “Changes in World View”, “Internal Changes” and “Growth from Patient Growth”. However, the Chinese version extracted four factors: “Professional Support and Growth”, “Interpersonal Empathy and Protection”, “Self-awareness and Sense of Worth” and “Personal Relations and Emotional Connection”.

These changes likely reflect cultural differences in how nurses experience post-traumatic growth. In the Chinese version, a new dimension, “Professional Support and Growth” was introduced, emphasizing the role of the workplace in fostering growth. This factor groups items related to work-life balance, emotional support from colleagues, and witnessing patient recovery, which were either not emphasized or were distributed across different factors in the English version. This shift suggests that Chinese nurses may experience post-traumatic growth more prominently through their professional interactions and the support they receive in the workplace, rather than solely through changes in worldview or self-perception.

In contrast, the English version’s “Internal Changes” dimension was divided into separate factors in the Chinese version. Items related to emotional resilience and personal values were relocated to the “Self-awareness and Sense of Worth” factor, while those concerning interpersonal empathy were grouped under “Interpersonal Empathy and Protection.” This restructuring indicates that, within Chinese culture, self-awareness and interpersonal relationships are viewed as distinct pathways to growth, highlighting the collectivist emphasis on harmony and relational well-being. The distinction between these factors underscores how Chinese nurses may prioritize empathy, protection of personal relationships, and the need for balance between personal and professional life when reflecting on trauma-related growth.

### Item reduction and reclassification

Validity refers to how accurately a research tool reflects the concept under study and its consistency with the underlying theory [48]. Reliability refers to the consistency and stability of results obtained from a measurement tool, including internal consistency and test-retest reliability [49]. The Chinese version of the VPTGI also involved a reduction in the number of items, from 32 in the original version to 22 in the final Chinese adaptation. This item reduction was driven by both psychometric and cultural considerations. During exploratory factor analysis (EFA), certain items showed similar loadings on multiple factors, blurring the distinction between latent variables. For example, items related to balancing work and life or processing emotions were found to overlap significantly, necessitating their removal to improve the model’s clarity and interpretability.

Additionally, some items were omitted or reclassified based on their cultural relevance. For instance, in Chinese culture, where emotional restraint and modesty are valued, items that emphasized open emotional expression were softened or excluded. The reclassification of items into the four-factor structure also reflects cultural shifts in the interpretation of growth, with greater emphasis placed on professional and interpersonal relationships as essential elements of post-traumatic growth. This highlights the importance of context in psychometric tool development, as direct translations may not fully capture the nuances of psychological constructs across cultures.

Despite the item reduction, the Chinese version maintained robust psychometric properties. The cumulative variance explained by the four factors was 76.134%, comparable to the original version, indicating strong structural validity. Moreover, the reliability of the Chinese version was confirmed, with Cronbach’s α coefficients for the four dimensions ranging from 0.795 to 0.936, and an overall Cronbach’s α of 0.965. These findings suggest that the revised Chinese VPTGI is a reliable and valid tool for assessing VPTG among Chinese nurses.

### VPTGI vs. PTGI: a critical advancement in measurement

Previous studies exploring the relationship between STS and growth often relied on the Posttraumatic Growth Inventory (PTGI), which was designed to measure growth in individuals directly exposed to trauma. While the PTGI captures various dimensions of post-traumatic growth (PTG), it was not specifically developed for assessing VPTG in helping professionals, such as nurses, who are indirectly exposed to trauma through their patients. As a result, the use of the PTGI in past research may have led to less precise assessments of the factors contributing to VPTG, potentially skewing findings and failing to fully capture the unique experiences of healthcare professionals.

In contrast, this study used the Vicarious Posttraumatic Growth Inventory (VPTGI), a tool specifically designed to measure the growth resulting from indirect trauma exposure through empathetic engagement. The VPTGI provides a more accurate assessment of how nurses experience growth in their professional roles, particularly in relation to their interactions with patients. By using the VPTGI, this study offers new, more targeted evidence on the relationship between STS and VPTG, providing a clearer understanding of how secondary trauma impacts both positive and negative outcomes in healthcare workers.

### The relationship between STS and VPTG: a new perspective

A objective of this study was to examine the relationship between STS and VPTG among Chinese nurses. Past studies have yielded inconsistent results regarding this relationship, with some suggesting that STS can coexist with growth, while others indicate that higher levels of stress may impede growth [24, 25, 50]. However, many of these studies relied on the PTGI, which may not have been appropriate for capturing the unique nature of VPTG.

In this study, we found a significant negative correlation between STS and VPTG (*r* = -0.021, *P* < 0.001). This suggests that higher levels of STS can hinder the development of VPTG, likely by overwhelming nurses’ emotional capacities and limiting their ability to engage in reflective practices that foster growth [51]. Unlike previous studies, which may have failed to detect this relationship due to the use of less specific tools, the use of the VPTGI in this study provides stronger and more reliable evidence of this inverse relationship.

Joseph’s theoretical framework posits that some level of stress is necessary for growth to occur, but excessive stress, as seen in STS, may obstruct growth by impairing coping mechanisms and emotional resilience [52]. This duality, where trauma exposure can simultaneously lead to both stress and growth, underscores the complexity of the STS-VPTG relationship. The use of the VPTGI in this study allowed for a more nuanced understanding of this relationship, demonstrating that while growth is possible, it requires the mitigation of secondary traumatic stress to foster optimal outcomes.

### Implications for practice and future research

The findings of this study have important implications for both research and clinical practice. First, the successful adaptation and validation of the VPTGI in the Chinese context provides a powerful tool for measuring growth in healthcare professionals exposed to indirect trauma. Unlike previous studies that relied on the PTGI, this study offers more accurate and culturally relevant insights into how Chinese nurses experience VPTG. The negative correlation between STS and VPTG underscores the need for interventions that not only reduce stress but also promote growth.

By using the VPTGI, this study advances the field by providing clearer evidence of the distinct relationship between stress and growth in helping professionals. Future research should continue to explore this relationship using the VPTGI in other cultural and professional settings to further validate its applicability and examine the long-term effects of interventions designed to reduce STS and foster VPTG. Additionally, psychological training and support programs should be developed to help nurses manage the emotional toll of indirect trauma while enhancing their capacity for growth.

### Limitations

First, although this study had an ample sample size, the participants were exclusively nurses from tertiary hospitals, which might introduce selection bias. Future research could consider expanding the sample size and conducting further investigations in hospitals of varying levels to mitigate this limitation. Second, the causal relationships among variables should be interpreted with caution due to the cross-sectional design of the study.

## Conclusion

The Chinese version of the VPTGI comprises four dimensions with a total of 23 items, presenting concise and clear expressions while retaining the conceptual integrity and semantic equivalence of the original scale. This scale’s content aligns with the cultural context and national conditions of China, demonstrating good validity and reliability. It serves as an effective tool for evaluating VPTG among nurses in China. The application of this iventory within the Chinese cultural context holds significant importance for promoting attention and research on nurses’ VPTG. This study also sheds light on the relationship between STS and VPTG within the context of China. It is recommended that future research should investigate the factors influencing these variables further to facilitate the reduction of STS and promotion of VPTG among nurses.

## Supplementary Information

Supplementary Material 1.

Supplementary Material 2.

Supplementary Material 3.

## Data Availability

The datasets referenced in this article are not readily available to the public, as they were generated and/or analyzed during the current study with the intention of preserving the anonymity of the respondents. However, these datasets can be obtained upon reasonable request from the corresponding author. Requests for access to the datasets should be directed to caiytlucky@163.com.

## Abbreviations

VPTG: Vicarious posttraumatic growth
PTG: Posttraumatic growth
VPTGI: Vicarious Posttraumatic Growth Inventory
PTGI: Posttraumatic growth Inventory
STS: Secondary Traumatic Stress
COSMIN: Consensus-based standards for the selection of health measurement instruments
