# Supplementary material for: Cross-cultural adaptation and reliability of the inventory of vicarious posttraumatic growth and research of its influencing factors: a cross-sectional study
Source: BMC Nurs. 2024 Oct 17;23:763. doi: 10.1186/s12912-024-02435-5 (PMC11487754; doi:10.1186/s12912-024-02435-5)
Supplement: Supplementary file 1 — Supplementary Material 1. [file 12912_2024_2435_MOESM1_ESM.pdf]

中南大学护理学院

护理与行为医学研究伦理审查委员会

项目名称：	护士替代性创伤后成长问卷的修订及替代性创伤后成长的相关影响因素的横断面调查		
项目负责人：	蔡依彤		
伦理评审编号：	E2023110	伦理评审日期：	2023年 03月 23日
伦理评审类型：	快速审查	评审有效期：	1年

经中南大学护理学院护理与行为医学研究伦理审查委员会审议，认为此项目符合伦理审查通过的要求，在上述有效期内伦理评审有效。

请在项目执行过程中严格遵守医学伦理道德原则，按照研究计划和申请报告进行，保障受试者的权益，并及时向本伦理委员会报告项目执行过程中发生的意外事件和处理情况。

中南大学护理学院护理与行为医学研究伦理审查委员会

2023年 03月 25日

中南大学护理学院行为医学与护理学伦理审查委员会

xyhllsc
