# Supplementary material for: Cross-cultural adaptation and reliability of the inventory of vicarious posttraumatic growth and research of its influencing factors: a cross-sectional study
Source: BMC Nurs. 2024 Oct 17;23:763. doi: 10.1186/s12912-024-02435-5 (PMC11487754; doi:10.1186/s12912-024-02435-5)
Supplement: Supplementary file 2 — Supplementary Material 2. [file 12912_2024_2435_MOESM2_ESM.docx]

Figure 1 Confirmatory factor analysis model of the revised Chinese version of the Nurse Vicarious Posttraumatic Growth Scale

A: Personal Growth and Care

B: Professional Balance and Inspiration

C: Professional Meaning and Self-Awareness

D: Development and Maintenance of Personal Relationships
