# Supplementary material for: Cross-cultural adaptation and reliability of the inventory of vicarious posttraumatic growth and research of its influencing factors: a cross-sectional study
Source: BMC Nurs. 2024 Oct 17;23:763. doi: 10.1186/s12912-024-02435-5 (PMC11487754; doi:10.1186/s12912-024-02435-5)
Supplement: Supplementary file 3 — Supplementary Material 3. [file 12912_2024_2435_MOESM3_ESM.docx]

Supplementary file

The original Chinese and English version of the VPTGI

| **维度** | **条目内容** |
| --- | --- |
| Negative Responses to Trauma Work 对创伤工作的消极反应 | 1. I have experienced negative emotional reactions as a result of hearing the trauma story (i.e. irritability, sadness, anger, or frustration) |
|  | 我因听到患者的创伤故事而经历过负面情绪反应（如烦躁、悲伤、愤怒或沮丧） |
|  | 2. I have experienced negative physical symptoms as a result of hearing the trauma story (i.e. headaches, sleep disturbance, nausea, or increased heart rate) |
|  | 我因听了患者的创伤故事而出现了负面的身体症状（如头痛、睡眠障碍、恶心或心率加快） |
|  | 3. I have questioned my ability to fulfill my role or scope of practice |
|  | 我曾质疑自己履行角色或执业范围的能力 |
|  | 4. There are horrifying trauma stories that I will never forget. |
|  | 有一些可怕的创伤故事，我一直无法忘记 |
|  | 5. I have experienced negative intrusive thoughts regarding my client or patients’ trauma experience. |
|  | 因为我的患者的创伤经历，我曾有过负面的侵入性思维 |
| Changes in World View 世界观的变化 | 6. I have a greater appreciation of my life experiences. |
|  | 我更感激我的生活经历 |
|  | 7. I seek understanding of other people and their experiences. |
|  | 我试图去理解他人及其经历 |
|  | 8. I am more knowledgeable and aware of social justice issues and concerns. |
|  | 我更了解和关心社会公正问题 |
|  | 9. I am less judgmental towards others. |
|  | 我对别人不那么挑剔了 |
|  | 10. I am more empathic towards others and their experiences. |
|  | 我对他人及其经历更有同理心 |
|  | 11. I have learned a perspective of others whom are different than me. |
|  | 我了解了其他人的不同观点 |
| Creating Meaning to Change Self 创造意义，改变自我 | 12. I have been inspired to overcome adversity in my own life. |
|  | 我受到了鼓舞从而克服自己生活中的挫折 |
|  | 13. I have gained a sense of value and importance in my work. |
|  | 我在工作中获得了价值感，体会到自己的重要性 |
|  | 14. I have a greater sense of purpose in life. |
|  | 我有更大的人生目标感 |
|  | 15. I am more self-aware. |
|  | 我有更强的自我意识（自我意识是对自身、他人、社会关系有正确认识的整体意识） |
|  | 16. I feel rewarded being in a needed profession. |
|  | 我觉得从事一个需要的职业很有成就感 |
| Changes in Interpersonal Relationships 人际关系的变化 | 17. I have a greater appreciation for my personal relationships who have not experienced trauma. |
|  | 我更加感激自己暂未经历过创伤的人际关系 |
|  | 18. I am more emotionally expressive in my relationships. |
|  | 我在人际关系中更善于表达自己的情感 |
|  | 19. I make more attempts to connect in my personal relationships. |
|  | 我更多地尝试建立人际关系 |
|  | 20. I express more kindness in my relationships. |
|  | 我在人际关系中表达更多的善意 |
|  | 21. I am protective of my personal relationships to prevent trauma. |
|  | 我对我的个人关系进行保护，以防止创伤 |
| Engaging in Efforts of Support and Self Care 支持和自我照顾的努力 | 22. I try to find my own ways to process the trauma of my clients or patients. |
|  | 我试图找到自己的方法来处理患者的创伤 |
|  | 23. I seek out ways to escape, or detach, from my work during my time off. |
|  | 在休息时间，我想方设法逃避，或脱离我的工作 |
|  | 24. I talk with others who understand my work. |
|  | 我与了解我工作的人交谈 |
|  | 25. I have established boundaries to support work life balance. |
|  | 我已经建立了维持工作生活平衡的界限 |
|  | 26. I seek support from colleagues to help me maintain my well-being. |
|  | 我寻求同事的支持来帮助我保持康乐 |
|  | 27. I put my thoughts and feelings aside in order to continue working. |
|  | 为了继续工作，我会把自己的想法和感受放在一边 |
| Client Progress Impacting Growth 患者改善促使的成长 | 28. Witnessing my client’s growth has led to feelings of hope in my own life. |
|  | 见证患者的成长让我对自己的生活充满希望 |
|  | 29. I respect the strength my clients or patients have had in recovering from trauma. |
|  | 我对我得患者从创伤中恢复的力量充满敬意 |
|  | 30. I am inspired by what my clients and patients have been through. |
|  | 我从患者的经历中受到了激励 |
|  | 31. As a result of my work, I believe people are able to overcome and move on from their past. |
|  | 由于我的工作，我相信患者能够克服过去并继续前进 |
|  | 32. My client(s) or patient(s) have who have experienced trauma have taught me something about myself. |
|  | 我的患者经历了创伤，让我对自己有了一些更深的了解 |

Chinese version of VPTGI

| 维度 | 条目 |
| --- | --- |
| Professional Support and Growth 职业支持与成长 | 24. I talk with others who understand my work. |
|  | 24. 我与理解我工作的人进行交流 |
|  | 25. I have established boundaries to support work life balance. |
|  | 25. 我能平衡工作和生活 |
|  | 26. I seek support from colleagues to help me maintain my well-being. |
|  | 26. 我寻求同事的支持来帮助自己保持良好的状态 |
|  | 27. I put my thoughts and feelings aside in order to continue working. |
|  | 27. 我会把自己的想法和感受放在一边，以便继续工作 |
|  | 28. Witnessing my client’s growth has led to feelings of hope in my own life. |
|  | 28. 见证患者的成长让我对自己的生活充满希望 |
|  | 29. I respect the strength my clients or patients have had in recovering from trauma. |
|  | 29. 我对从创伤中恢复的患者充满敬意 |
|  | 30. I am inspired by what my clients and patients have been through. |
|  | 30. 我从患者的经历中受到了激励 |
| Interpersonal Empathy and Protection 人际共情与保护 | 9. I am less judgmental towards others. |
|  | 9. 我对他人更加宽容了 |
|  | 10. I am more empathic towards others and their experiences. |
|  | 10. 我对他人及其经历更有同理心 |
|  | 11. I have learned a perspective of others whom are different than me. |
|  | 11. 我已经学会了采取不同的角度去看待与我观点不同的人 |
|  | 12. I have been inspired to overcome adversity in my own life. |
|  | 12. 那些经历过创伤的病人激励我去克服生活中的挫折 |
|  | 20. I express more kindness in my relationships. |
|  | 20. 我在人际交往中表达出更多的善意 |
|  | 21. I am protective of my personal relationships to prevent trauma. |
|  | 21. 我保护我的私人人际关系以避免创伤 |
|  | 22. I try to find my own ways to process the trauma of my clients or patients. |
|  | 22. 我试图找到自己的方法来应对患者的创伤 |
| Self-Awareness and Sense of Worth 自我认知与价值感 | 6. I have a greater appreciation of my life experiences. |
|  | 我更感激我的生活经历 |
|  | 13. I have gained a sense of value and importance in my work. |
|  | 我在工作中获得了价值感，体会到自己的重要性 |
|  | 14. I have a greater sense of purpose in life. |
|  | 我有更大的人生目标感 |
|  | 15. I am more self-aware. |
|  | 我有更强的自我意识（自我意识是对自身、他人、社会关系有正确认识的整体意识） |
|  | 16. I feel rewarded being in a needed profession. |
|  | 我觉得从事一个需要的职业很有成就感 |
| Personal Relations and Emotional Connection 私人人际与情感维系 | 17. I have a greater appreciation for my personal relationships who have not experienced trauma. |
|  | 我更加感激自己暂未经历过创伤的人际关系 |
|  | 18. I am more emotionally expressive in my relationships. |
|  | 我在人际关系中更善于表达自己的情感 |
|  | 19. I make more attempts to connect in my personal relationships. |
|  | 我更多地尝试建立人际关系 |
